# Attendance in a Neonatal Follow-Up Program before and in the Time of COVID-19 Pandemic: A Mixed Prospective–Retrospective Observational Study

**DOI:** 10.3390/children11091138

**Published:** 2024-09-19

**Authors:** Evdoxia Nantsi, Ilias Chatziioannidis, Abraham Pouliakis, Georgios Mitsiakos, Elias Kondilis

**Affiliations:** 1 Laboratory of Primary Health Care, School of Medicine, General Medicine and Health Services Research Aristotle University, 54124 Thessaloniki, Greece; evdoxia_nantsi@hotmail.com (E.N.); ekondilis@auth.gr (E.K.); 2 Second Neonatal Department and Neonatal Intensive Care Unit (NICU), Aristotle University of Thessaloniki, “Papageorgiou” University Hospital, 56403 Thessaloniki, Greece; mitsiakos@auth.gr; 3 Second Department of Pathology, National and Kapodistrian University of Athens, “Attikon” University Hospital, 12462 Athens, Greece; apouliak@med.uoa.gr

**Keywords:** attendance, neonate, follow up program, COVID-19 pandemic

## Abstract

Background: Attendance to neonatal follow-up programs presents a significant factor associated with positive long-term outcomes of high-risk infants. Strategies to maximize participation benefit not only future interventions’ effectiveness but also healthcare systems and society. While a number of studies have focused on attrition or loss to follow-up, no studies have focused on the contributive risk factors to abstaining from neonatal follow-up programs specifically during the COVID-19 pandemic. This study aims to reveal the main factors linked to non-compliance in a neonatal follow-up program of a tertiary hospital. Methods: In this ambidirectional observational study, data from 1137 high-risk neonates who participated in a hospital follow-up program were collected (573 before and 564 after the COVID-19 pandemic). The study sample was grouped to three groups: G1 (N = 831), who maintained participation in the program; G2 (N = 196), who discontinued; and G3 (N = 110), who never visited the outpatient clinics. Data were obtained from the hospital’s Systems Applications and Products (SAP) Software and a structured questionnaire, answered by parents of newborns either discontinuing (G2) or not attending (G3) the follow-up program through a telephone contact. Results: The most frequently reported reason for discontinuance before the pandemic onset was the parents’ perception of no necessity to maintain participation (44.12%). During the COVID-19 pandemic, provider-related barriers to maintaining hospital access, inability to provide high-quality services (37.14%), and feelings of fear and insecurity (18.5%) emerged as factors for non-attendance. Citizenship and morbidity (respiratory distress syndrome, sepsis, necrotic enterocolitis, jaundice) acted as incentives to join the follow-up program during both study periods. Multiple regression analysis showed that multiple-gestation infants had higher odds of maintaining participation during the COVID-19 period (OR, 4.04; CI, 1.09–14.9). Conclusions: Understanding the potential impact of COVID-19 and the transformative changes in neonatal follow-up clinics is crucial for applying compliance strategies. Removing barriers to maintain family participation can lead to increased attendance rates.

## 1. Introduction

The World Health Organization estimates premature births at 15 million worldwide, with varying rates from 5% to 18% of all births [1,2]. Premature infants require long-term hospitalization, and neonatal follow-up (NFU) programs are essential to prevent serious health problems that may affect their later life [3,4,5]. NFU programs play an integral and crucial role in long-term care of high-risk infants, focusing on neurodevelopmental impairment [6,7,8,9], family counseling and support, optimal and continuous monitoring for health problems, and early intervention [4,9]. High-risk neonates for NFU are pre-emies (≤32 weeks and/or ≤1500 g), with hypoxic–ischemic encephalopathy, convulsions, Central Nervous System (CNS) disorders, congenital infections, genetic syndromes, MRI pathological results, major morbidities, shock requiring inotropic support, severe hypoxemic episodes, hypoglycemia, hyperbilirubinemia treated with exchange blood transfusion, Small for Gestational Age (SGA), mechanical ventilation support > 24 h, feto–fetal transfusion, metabolic diseases, abnormal neurological examination at discharge, surgical treatment during Neonatal Intensive Care Unit (NICU) hospitalization, and low socio-economic family status [8,10]. The interdisciplinary teams providing NFU programs mainly consist of Neonatologists, Physiotherapists, and Speech Therapists [10,11].

Attrition or loss to NFU has a significant impact on the long-term health and, specifically, the neurodevelopmental outcome of high-risk infants [12,13,14,15,16,17]. Consequently, reasons for non-participation in NFU programs [6,18] and strategies implemented for achieving higher attendance rates are of major importance [19]. Several studies highlight the importance of defining reasons for non-attendance in such programs, as well as planning and implementing certain strategies [6,18,20,21,22,23,24,25,26,27,28,29]. The COVID-19 pandemic caused a decline in access to health services [30], leading to limitation or even interruption of service provisioning, and even an increase in queueing time for prevention and rehabilitation health care [30,31]. The Greek health system was not an exception, as it received enormous pressure [32]. This pandemic had a severely negative impact on maternal and infant health care programs [33,34], highlighting the necessity of defining strategic plans both at the national and international level to restore the pre-pandemic levels of health services [35]. To date, there is no published study investigating potential changes in reported reasons for non-attendance before and after the spread of COVID-19 and the impact on access to NFU programs. 

This study aims to investigate the impact of the COVID-19 pandemic on access to a NICU NFU program, to determine parent-reported reasons for non-attendance and predictors for non-compliance. Additionally, the main factors linked to non-compliance leading before and after the onset of COVID-19 pandemic were investigated. We hypothesized that attendance rates and reasons for discontinuation of the NFU program could differ before and during the COVID-19 pandemic. 

## 2. Materials and Methods

We conducted a mixed prospective and retrospective observational study using a questionnaire to collect data from families whose newborns were scheduled to participate in a NFU program at the Papageorgiou Hospital Outpatient Clinics before and during the SARS-CoV-2 pandemic in Greece. The study hospital is tertiary, and the level III NICU has, on average, 548 admissions/per year. The data for this study were obtained from two sources: firstly, from the hospital’s Systems Applications and Products Software (SAP) (ECC 6), which provided data of high-risk infants hospitalized in the NICU, and secondly, through a structured questionnaire, answered by parents of newborns either discontinuing or not attending the NFU program through telephone contact. The researcher (E.N.) tried to contact parents who were non-consistent to the scheduled appointments by phone. All parents who answered the researcher’s calls agreed to participate in the research and were included in the research. Parents who did not respond to three phone-call attempts were excluded from the survey. The questionnaire consisted of closed-ended questions, some of which allowed multiple answers, as well as open-ended questions. The closed-ended questions focused on participation in the monitoring program, while the open-ended questions focused on factors that influenced parents during the visits (Appendix A).

In particular, demographic characteristics of the participating families were obtained from the SAP and questionnaires. This information included the age of the father/mother, number of adults/children in the family, single parenthood, working hours, work status of the caregiver, daily work of the caregiver, education level, gender of the research participant, number of visits to the Hospital’s Outpatient Clinic, nationality, and hospital accessibility data, such as distance, transport time, and number of tolls. The hospital’s information system (SAP) was used to search and retrieve information notes for the newborns, when available. This allowed for the recording of various clinical characteristics of the perinatal period. Specifically, the following parameters were recorded: birth weight (BW), gestational age (GA), method of delivery (vaginal or cesarean section), gender, mother age, multiple births, gestational diabetes, hypertension, antenatal steroid administration (ASA), multiparity, intrauterine growth restriction (IUGR), presence of early or late septicemia, and jaundice. Additionally, major morbidity factors such as Respiratory Distress Syndrome (RDS), bronchopulmonary dysplasia (BPD), necrotizing enterocolitis (NEC), and retinopathy of prematurity (ROP) were also recorded.

Before conducting the research, permission was requested and obtained from Papageorgiou Hospital (reference code: 118/21-04-2021), and approval was obtained from the Ministry of Health. Anonymity was ensured, and participants were informed of their right to recall their data. Finally, the researcher digitally forwarded the consent form to the participants.

### 2.1. Data Collection

The sample comprises two groups of mother–infant pairs. The first group involved newborns who were monitored before the onset of the COVID-19 pandemic, with at least one scheduled appointment between 12 February 2019 and 29 February 2020 (with no appointments scheduled beyond 29 February 2020, i.e., the date that measures for citizen protection against COVID-19 were initiated in Greece). The second group involved newborns after the onset of the COVID-19 pandemic who had at least one scheduled appointment between 3 January 2020 and 17 November 2021. Each group was divided into three additional groups: Group G1, with families of neonates who did not miss any scheduled visit; Group G2, for families that visited the outpatient clinic at least once and subsequently discontinued follow-up; and Group G3, for families that never visited the outpatient clinics. The reported reasons that led participants to discontinue follow-up were grouped by relevance, as shown below (Table 1).

### 2.2. Statistical Analysis

The data were collected in Excel spreadsheets, and statistical analysis was performed using SAS for Windows (SAS Institute Inc., Cary, NC, USA) statistical analysis software (version 9.4). Descriptive statistics for the arithmetic data were presented as median and 1st–3rd quartiles (Q1–Q3), while normality was evaluated by the Kolmogorov–Smirnov test. For categorical data, descriptive statistics are presented using frequency and relevant percentages. For comparisons between groups: for arithmetic data, the Mann–Whitney U test or the Kruskal–Wallis test was used, the latter for more than two groups; for categorical data, the chi-square test and, if required, Fisher’s exact test, were used. Multivariable analysis was based on logistic regression models. The significance level for the study was set to *p* < 0.05, and all tests were two-sided.

## 3. Results

**Scheme 1:**
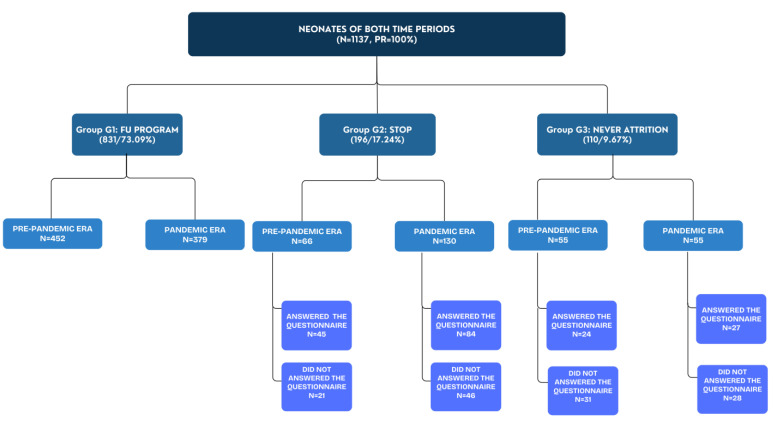
Parents of infants who answered the questionnaire by period.

In total, a sample of 1137 high-risk newborns with 2254 scheduled appointments was recorded. Particularly, 831 newborns (73.08%) consistently participated in the follow-up program (group G1), while 196 (17.24%) discontinued the follow-up (group G2), and 110 (9.67%) did not attend any planned visits (group G3) (Scheme 1). Out of 306 parents who discontinued or did not attend the NFU program (group G2 + G3), 180 responded to the questionnaire (total response rate 58.8%), 69 from the pre-COVID-19 time period and 111 from the COVID-19 time period (Scheme 1).

During the study period before the onset of the COVID-19 pandemic, 573 newborns were recorded, with 452 included in the G1 group, 66 in the G2 group, and 55 in the G3 group (Scheme 1). After the onset of the COVID-19 pandemic, 564 neonates were recorded, with 379 included in the G1 group, 130 in the G2 group, and 55 in the G3 group (Scheme 1). Additionally, before the onset of the COVID-19 period, 1132 scheduled appointments were recorded, with 143 (12.6%) not being carried out, either being postponed or “lost”. During the COVID-19 period, 1122 scheduled appointments were recorded, with 323 (28.8%) not being carried out, either being postponed or “lost”. It is important to acknowledge that outpatient clinics were universally closed for 5.5 months during the pandemic [32]. Therefore, the number of appointments in the COVID-19 period were less than those carried out in the previous period.

### 3.1. Clinical and Demographic Characteristics before and after the Onset of COVID-19

Birth weight of newborns with scheduled visits after the onset of COVID-19 pandemic was found lower by an average of 260 g than those before the pandemic (*p* = 0.0005). Furthermore, infants with scheduled visits during the COVID-19 pandemic had a median gestational age of 33.1 weeks (Q1–Q3: 31.4–35.7), while those who attended before the COVID-19 pandemic had a median gestational age of 34.6 weeks (Q1–Q3: 32–37.3), indicating a reduction in pregnancy duration by 3.5 weeks. Moreover, gestational diabetes and RDS were more frequent in the group of infants after the onset of the COVID-19 pandemic. On the other hand, a higher percentage of infants developed early onset sepsis before the onset of the COVID-19 pandemic (Table 2).

### 3.2. Attendance Rates before and after the Onset of COVID-19

During the study time period, 989 scheduled appointments were completed at outpatient clinics before the COVID-19 pandemic (from 12 February 2019 to 29 February 2020, i.e., 12.5 months) and 799 after COVID-19 onset (from 1 March 2020 to 17 November 2021, i.e., 15.5 months). Additionally, 28.8% of appointments scheduled in the COVID-19 period were postponed or did not take place; this percentage is significantly higher than the percentage of the period before the onset of the pandemic (12.6%).

#### 3.2.1. Reasons for Non-Attendance

With regard to the G2 group, during the pre-COVID-19 period, the main reported reasons for discontinuing the NFU program were the perception that it was not necessary to maintain participation in the program (R6), at a rate of 44.12%, provider-related barriers (R4) at 26.47%, and accessibility-related barriers (R2) and negligence (R5) at 11.76%. In addition, the parents did not mention any health issue or death in the family (R7) or insecurity feelings about the hospital environment (R8). In the COVID-19 period, provider-related obstacles (R4) at 37.14%, fear of risk exposure (R8) at 18.57%, and health issues or death in the family (R7) at 15.71% emerged as the main reasons.

**Figure 1:**
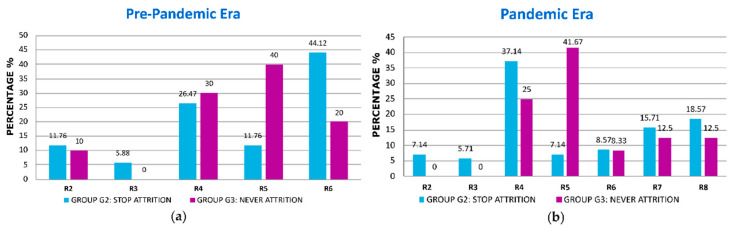
The incidence of the various reported causes that led to either cessation of participation (group G2) or total abstinence (group G3): (**a**) in the pre-COVID-19; (**b**) in the COVID-19 time period.

With regard to the G3 group, reasons for non-participating at the NFU program during pre-COVID-19 period were negligence (R5) at 40%, provider-related obstacles (R4) at 30%, and a perception that it was not necessary to maintain participation in the program (R6) at 20%. In addition, in the COVID-19 period, parental negligence (R5) at 41.67%, provider-related obstacles (R4) at 25%, and health issues (R7) and fear of disease exposure (R8) at 12.5% were the main reasons (see Figure 1).

#### 3.2.2. Comparisons between Groups before the Onset of COVID-19

In order to identify factors related to NFU program participation for the period before COVID-19, we compared all three groups (G1, G2, and G3). Significant differences in birth weight, gestational age, antenatal steroid administration, multiparity, jaundice, respiratory distress syndrome, and nationality were found (Table 3). Specifically, morbidity (hyperbilirubinemia (OR, 2.7; CI, 1.47–5, *p* = 0.0016), RDS (OR, 2.33; CI, 1.02–5.26, *p* = 0.00493)), ASA (OR, 4.76; CI, 1.37–16.67, *p* = 0.007), multiple gestation (OR, 2.94; CI, 1.35–6.25, *p* = 0.0047), and nationality (OR, 3.13; CI, 1.56–6.25, *p* = 0.0037) acted as incentives for participating in outpatient clinics of the NFU program (Table 3). Of note, infants who discontinued the NFU program (group G2) were the most immature (gestational age 32.9 (29.7–35.6) weeks) and of the lowest BW (1750 (1355–2400 gr)) (Table 3).

#### 3.2.3. Comparisons between Groups after the Onset of COVID-19

Regarding the COVID-19 time period, comparison between three groups showed a statistically significant difference for morbidities (LOS, NEC, Jaundice, RDS) (Table 4). Particularly, when comparing groups G1 and G2, LOS (OR, 0.48; CI, 0.28–0.82, *p* = 0.078) and NEC (OR, 0.32; CI, 0.11–0.93, *p* = 0.0467) did not present incentives for program completion; in contrast, they appeared to have a positive effect on joining the NFU program but not on maintaining participation in it. The comparison of groups G1 and G3 showed that more immature neonates, of lower birth weight with morbidities (jaundice (OR, 3.45; CI, 1.82–6.67, *p* = 0.0001), LOS (OR, 4.17; CI, 0.97–16.67, *p* = 0.0372), and RDS (OR, 2.44; CI, 1.2–5, *p* = 0.0155)) participated the NFU program.

#### 3.2.4. Multiple Regression Analysis

Multiple regression analysis showed that multiple-gestation birth was the sole significant factor in continuing the NFU program in the COVID-19 period. Neonates of multiple pregnancies had higher odds to continue the NFU during the COVID-19 period (OR, 4.04; CI, 1.09–14.9). Additionally, before the COVID-19 period, no factors were found as significant to lead towards participation in the NFU program.

## 4. Discussion

Improving attendance to NFU programs and addressing barriers to participation are key priorities for enhancing the quality of neonatal care [6,36,37,38]. However, the impact of the COVID-19 pandemic on access to NFU programs and the potential barriers for family participation have not been thoroughly studied. Our research reveals significant differences in the reasons for participation in an NFU program before and after the onset of the pandemic, highlighting the need for further exploration. Additionally, morbidities (RDS, jaundice, NEC, LOS), ASA, multiple gestation, and nationality were significant factors for participation in the NFU program.

The study of Kondilis et al. reveals that the utilization of essential public hospital services in Greece during the COVID-19 pandemic (from January to November 2020) compared to the 2017–2019 period decreased significantly [32]. Specifically, visits to non-emergency public hospital departments decreased by 33.3% (95% CI: 16.0%–50.5%) [32]. In our study, the non-attendance rate increased significantly by 2.5-fold during the COVID-19 pandemic (28.8% compared to 12.6%). This is also in line with the study by Panda et al. (2021), which underlines that in the USA, 43% of programs showed a decrease in attendance rates even one year after the onset of COVID-19 pandemic [39].

Newborns that did not attend any visits (G3 group) before and after the onset of COVID-19 tended to be more mature and healthier (without morbidities: RDS, jaundice, NEC, LOS), without ASA, and they were mostly from immigrant families with more children. Moreover, newborns that attended the program but did not continue follow up (group G2) had morbidities such as LOS or NEC, whose existence appears to have a positive effect on joining the NFU program but not on maintaining participation.

Most of the findings of this research do support already-existing international literature findings. Researchers have shown that families with clinically ill neonates during hospitalization were more likely to adhere to an NFU program [20,22,26,27,28,40]. According to the research of Patra et al., very low birth weight, small gestational age, multiple gestation, and Chronic Lung Disease were factors significantly associated with follow-up visits attendance [6]. Additionally, Harmon and Sweringen et al. showed that longer durations of oxygen hospitalization and mild chronic lung disease were associated with greater and lower odds of NFU compliance, respectively [20,22]. Moreover, in the study by Swearingen et al. an inverse relationship of neonates’ gestational age with participation in the program was shown, findings similar with the present research results [22]. Of note, Perenyi et al. comments that parents of neonates with severe health issues or longer hospital stay have more opportunities to understand their infant’s condition, leading to better compliance [17]. Additionally, neonates of singleton pregnancies were four times more likely to discontinue their participation in the follow-up program after the onset of COVID-19. This is in line with the study of Kim et al., which suggests that the attendant group had higher rates of multiple pregnancies [28]. In our study, parents of infants of multiple-gestation pregnancies reported challenges in accessing health care during the COVID-19 pandemic. They mentioned difficulties with rapid testing and restrictions regarding the number of family members allowed during follow-up visits. It is important to note that several parents from group G2 highlighted these difficulties during their waiting time in the hospital’s outpatient clinics. Despite these challenges, they emphasized the significance of monitoring their infants, as they considered the multiple gestation as a “case at higher risk”. Most parents admitted feelings of anxiety about their newborns’ health due to high morbidity risk and low birth weight. Indeed, the birth weight of neonates of multiple gestation pregnancies (twins and triplets, birth weight: 1728 ± 497, n = 201), was significantly lower in comparison to singleton births (birth weight: 2247 ± 892, n = 466). During the period before the onset of the pandemic, the main reported reason for discontinuing follow-up (group G2) was the perception that it was not necessary to maintain participation in the program (R6), while the second-most common reason was related to obstacles caused by the provider (R4). Conversely, the main reason for stopping follow-up in the period after the onset of the pandemic was barriers related to the provider (R5), while the second-most common reason was fear of exposure to disease (COVID-19 or other diseases) (R8), followed closely by illness (or death) within the family (R7). With regard to the G3 group, the main reported reason for abstaining from the NFU program both in the period before and after the onset of the pandemic was parental neglect (R5). The second main reason for abstaining from the NFU program was various barriers related to the provider itself (R4) in both periods. Notably, references to a busy parental/family work schedule (R3) as a cause did not significantly differ between the two time periods.

The perception that participation in the NFU is unnecessary if their newborn does not develop health problems has also been reported as a reason for avoiding appointments (R6) by Ballantyne et al. and Duarte et al. [24,25]. Additionally, Tang et al. highlight key barriers to family attendance, such as parental work schedules (R3), the parent’s perceptions of the child’s health status, and lack of follow-up (R6) [18]. Swearingen et al. found scheduling issues on the part of providers as the second-most frequently reported barrier to participation (R4) [22]. Also, accessibility and distance from the clinic (R2) were highlighted as barriers to attendance by Tang et al. and Ballantyne et al. [18,25]. In the present research, barriers related to access and distance from the hospital were identified mainly before the onset of COVID-19 time period.

Also, the DeMauro et al. suggest that larger households are more likely to leave the program [23], while Ballantyne et al. [25] suggest that limited financial resources act as a barrier to participation in the program. As previously reported, larger families did not participate in the NFU program, mainly before the COVID-19 period.

Several studies have emphasized that difficulties with insurance coverage (R1) are a key factor leading to non-adherence to the follow-up program [6,18,20,40]. Fortunately, in this survey, none of the participating parents mentioned insurance coverage as a barrier to maintaining their participation.

Several studies underline the role of telemedicine for multidisciplinary developmental follow-up after NICU discharge, in comparison to in-person meetings [23]. Watson et al. showed that telemedicine was associated with an increased attendance rate in a twelve-month follow-up program for preterm infants [41]. In contrast to other similar NFU programs in other countries, such as the USA, Canada, and Italy, which tried to exploit the potential benefits of telemedicine by conducting remote appointments with the participation of multidisciplinary teams during the pandemic [39,42], in the current NFU program, a similar approach was not implemented. Of note, our NICU did not develop any strategy of enhanced support for parents who faced difficulties attending the program. This lack of a specific strategy for attending an NFU program has also been referred to by other studies. Tang et al. indicated that 54% of NFU programs lack a specific strategic plan for improved attrition [18].

It is of extreme importance for NICU employees (physicians, nurses, and administrative staff) to effectively communicate with parents about the importance of the NFU program and the potential benefits of maintaining participation in the program. In conclusion, staff members should encourage the parents to be in contact for any difficulties, concerns, or questions about the follow-up schedule. This will allow appointments to be rescheduled immediately and prevent low attrition rates. Finally, continuous feedback for the barriers faced by the parents should be provided, so that any adopted strategies can be redefined by the provider as needed [43].

### Limitations

There are some limitations to be underlined. Firstly, some of the patients’ electronic records were incomplete or missing. Secondly, researchers collected only the clinical data available in the SAP digital system. Consequently, the present research did not examine specific clinical variables during hospitalization (oxygen administration, oxygen at discharge, length of stay, surgery requirement, breast feeding, and maternal drug use). Thirdly, the researchers could not investigate the reasons for non-adherence or abstention from the follow-up program in refugee population. Of note, the outdated contact information in the SAP system along with country relocation for several families made accessing this group impossible.

## 5. Conclusions

In conclusion, this study has revealed several key factors associated with adherence to an NFU program during the pre- and COVID-19 periods. It is imperative to implement targeted strategies such as patient education, reminder phone calls, and a systematic approach to increase attendance rate in NFU programs. These measures should be focused on families at risk for non-compliance. All these factors significantly contribute to better long-term patient care and neurodevelopmental outcomes through early detection and intervention programs.

## Figures and Tables

**Table 1:** Categories of reasons for discontinuation of follow-up.

Reasons for Νon-Attendance (R)	Categorization
R1	Lack of health insurance.
R2	Accessibility (distance of the clinic from the permanent residence/lack of means of transport/change in residence/moving (to another region/country)/bad weather.
R3	Loaded parental/family work schedule.
R4	Obstacles related to hospital services (limited availability during clinic hours/difficulty with parking/long waiting time/bad communication with the secretariat/disappointment from the experience at the hospital. Change (cancelation or postponement) either after consultation (with the Hospital/with Papageorgiou’s doctors) or at the initiative of the parents, closed outpatient clinics due to COVID-19.
R5	Negligence–Absence of Specific Reason.
R6	Assessment by the parents that monitoring/FU appointment was unnecessary.
R7	Health issues/Death in the family.
R8	Fear of diseases exposure (to COVID-19 or other diseases)/Difficulties due to COVID-19 (“Because only one parent was allowed to accompany” or difficulty with Rapid Tests).

**Table 2:** Clinical and demographic characteristics of infants by period (Data Source: SAP System).

Characteristics	Pre-Pandemic Era (n = 573)	Pandemic Era (n = 564)	*p*	OR and 95% CI
Gender (Male) ^a^	315 (54.97)	283 (50.18)	0.1091	0.82 (0.65–1.04)
Birth weight (gr) ^a^	2090 (1620–2830)	1830 (1430–2490)	0.0005	NA
Gestational age (weeks) ^a^	34.6 (32–37.3)	33.1 (31.4–35.7)	0.0003	NA
Age of mother ^a^	32 (29–38)	33 (29–38)	0.3454	NA
Multiparity ^a^	1 (1–2)	1 (1–2)	0.4379	NA
Multiple Gestation ^b^	104 (34.9)	137 (34.25)	0.8724	0.97 (0.71–1.33)
Small for Gestational Age (SGA) ^b^	72 (25.09)	80 (20.89)	0.2256	0.79 (0.55–1.13)
Gestational Hypertension ^b^	30 (11.03)	55 (14.82)	0.1947	1.4 (0.87–2.26)
Gestational Diabetes ^b^	34 (13.08)	127 (36.6)	<0.0001	3.84 (2.52–5.85)
IntraUterine Growth Retardation (IUGR) ^b^	54 (19.85)	94 (25.34)	0.1078	1.37 (0.94–2)
Delivery mode: Cesarean Section (CS) ^b^	235 (80.48)	317 (83.42)	0.3607	0.82 (0.55–1.22)
Antenatal Steroid Administration (ASA) ^b^	62 (26.72)	137 (37.53)	0.0075	1.65 (1.15–2.36)
Respiratory Distress Syndrome ^b^	87 (29.49)	173 (42.3)	0.0005	1.75 (1.28–2.41)
Bronchopulmonary dysplasia ^b^	29 (9.83)	44 (10.76)	0.7092	1.11 (0.67–1.81)
Early onset sepsis (<=3 DOL) ^b^	35 (12.07)	13 (3.32)	<0.0001	0.25 (0.13–0.48)
Late onset sepsis (>3 DOL) ^b^	38 (13.1)	73 (18.39)	0.0742	1.49 (0.98–2.29)
Jaundice ^b^	236 (64.13)	277 (63.68)	0.9412	0.98 (0.73–1.31)
Necrotizing Enterocolitis ^b^	6 (2.08)	15 (3.87)	0.2623	1.9 (0.73–4.95)
Retinopathy of Prematurity ^b^	7 (2.45)	8 (2.04)	0.7941	0.83 (0.3–2.31)
Number of visits ^a^	1 (1–4)	2 (1–3)	0.8534	NA
Nationality (Foreign) ^b^	64 (11.17)	67 (11.88)	0.7114	0.93 (0.65–1.34)

^a^ Data presented as median (range). ^b^ Data presented as n (%). NA: Not Applicable.

**Table 3:** Comparison of all 3 groups (G1, G2, G3) in the pre-COVID-19 time period.

Characteristics	G1 Group (n = 451)	G2 Group (n = 66)	G3 Group (n = 53)	G1 vs. G2		G1 vs. G3	
				*p*	OR (95% CI)	*p*	OR (95% CI)
Number of visits ^a^	2 (1–5)	2 (1–3)	0 (0–0)	0.329605	NA	<0.0001	NA
Birth weight (gr) ^a^	2115 (1620–2835)	1750 (1355–2400)	2282.5 (1917.5–3290)	0.008906	NA	0.0264	NA
Gestational age (weeks) ^a^	34.6 (32.1–37.1)	32.9 (29.7–35.6)	36.3 (33–38.3)	0.002245	NA	0.0108	NA
Antenatal Steroid Administration (ASA) ^b^	43 (29.25)	14 (30.44)	3 (8.11)	0.86	1.06 (0.51–2.18)	0.0097	4.76 (1.37–16.67)
Multiparity ^a^	1 (1–2)	1 (1–2)	2 (1–2.5)	0.422272	NA	<0.0001	NA
Multiple Gestation ^b^	71 (38.17)	22 (37.93)	9 (17.31)	1	0.99 (0.54–1.82)	0.0047	2.94 (1.35–6.25)
Jaundice ^b^	169 (67.33)	43 (69.36)	23 (43.4)	0.8796	1.1 (0.6–2)	0.0016	2.70 (1.47–5)
Respiratory Distress Syndrome ^b^	54 (29.67)	23 (38.98)	8 (15.38)	0.2005	1.51 (0.82–2.79)	0.0493	2.33 (1.02–5.26)
Nationality (Foreign) ^b^	42 (9.31)	9 (13.64)	13 (24.53)	0.2706	0.65 (0.3–1.41)	0.0037	3.13 (1.56–6.25)

^a^ Data presented as median (range). ^b^ Data presented as n (%). NA: Not Applicable.

**Table 4:** Comparison of all 3 groups (G1, G2, G3) in the COVID-19 period.

Characteristics	G1 Group (n = 451)	G2 Group (n = 66)	G3 Group (n = 53)	G1 vs. G2		G1 vs. G3
				*p*	OR (95% CI)	*p*
Number of visits ^a^	2 (1–4)	2 (1–3)	0 (0–0)	0.0006	NA	0.05
Birth weight (gr) ^a^	1830 (1420–2430)	1710 (1410–2250)	2142.5 (1600–2660)	0.3045	NA	0.0373
Gestational age (weeks) ^a^	33.3 (31.1–36)	32.4 (30.9–34.7)	34.4 (33–37.4)	0.1450	NA	0.0030
Multiparity ^a^	1 (1–2)	2 (1–2)	2 (1–3)	0.1980	NA	0.0091
Jaundice ^b^	183 (68.03)	76 (63.87)	18 (38.3)	0.4833	NA	0.0001
Late onset sepsis (>3 DOL) ^b^	37 (16.02)	34 (28.33)	2 (4.35)	0.0100	0.48 (0.28–0.82)	0.0372
Necrotizing Enterocolitis ^b^	6 (2.64)	9 (7.76)	0 (0)	0.0467	0.32 (0.11–0.93)	0.5936
Respiratory Distress Syndrome ^b^	108 (44.81)	53 (44.17)	12 (25)	1	NA	0.0155
Hours of work ^b^	40 (20–40)	40 (0–40)	17.5 (0–40)	0.2736	NA	0.0239

^a^ Data presented as median (range). ^b^ Data presented as n (%). NA: Not Applicable.

## Data Availability

The data presented in this study are available upon request from the corresponding author.

## Appendix A

Questionnaire

Research: Attendance in a neonatal follow up program before and in the time of COVID-19 pandemic

1. Participant Code (filled in by the researcher): _________________________

Demographics

2. Guardian Gender (Mark only one answer): Man____ → Woman____

3. Age:____________

4. Nationality:____________

5. Marital Status (Mark only one answer): With a partner____ Without a partner____

6. Number of adults living together at home (and taking care of the newborn): __________

7. Number of Children: ____________

8. Professional Status: ________________________

9. Hours of work per week: ____________

10. Work on a daily basis (Mark only one answer): Yes ____ No ____

11. Educational level: _______________________

12. Place of permanent residence: ________________________

Special Questions (Choose the answer that best suits your situation)

13. Was there a recommendation from the pediatrician to stop the program/referral to another clinic? (Mark only one answer): Yes ____ No ____

14. We skipped the visit, due to:

(You can highlight more than one answer and prioritize them.)

_____ lack of insurance coverage

_____ the distance of the clinic from the permanent residence

_____ lack of means of transport

_____ change in residence/move (to another region/country)

_____ a busy parental/family work schedule

_____ limited availability during clinic hours

_____ that I/we judged that it was not necessary, our child was fine

_____there was no specific reason

_____ Other reason

15. If you answered “other” in the previous question, please briefly state the reason that led to the omission of the visit?

______________________________________________________________________________________________________________________________________________________________________

16. Is there anything in particular that made your experience participating in the follow-up program difficult or challenging that you would like recorded?

______________________________________________________________________________________________________________________________________________________________________
